# Multidrug-resistant Enterobacterales in commercial laying and free-range hens (*Gallus gallus domesticus*) under semiarid conditions of the Caatinga biome

**DOI:** 10.1007/s42770-026-02004-9

**Published:** 2026-07-01

**Authors:** Katianny Bezerra de Medeiros, Ana Beatriz Monteiro de Medeiros, Débora Luise Canuto de Sousa, José Diniz de Souto Sobrinho, Tiago Casella, Carolina Sousa Américo Batista de Santos, Sérgio Santos de Azevedo

**Affiliations:** 1 Postgraduate Program in Animal Science and Health, Federal University of Campina Grande, Patos, PB Brazil; 2 Faculty of Medicine of São José do Rio Preto - FAMERP, São José do Rio Preto, São Paulo, Brazil

**Keywords:** Poultry, Antimicrobial resistance, Enterobacterales, Extended-spectrum beta-lactamases, One health

## Abstract

Antimicrobial resistance (AMR) is considered one of the greatest challenges to global health and a direct threat to human, animal, and environmental health. Thus, the objective of this survey was to characterise the antimicrobial susceptibility profile of Enterobacterales isolated from *Gallus gallus domesticus* (commercial laying hens [CLH] and free-range hens [FRH]) in the Caatinga biome. Cloacal swabs were collected from 165 CLH and 165 FRH in the states of Paraíba and Rio Grande do Norte, covered by the Caatinga biome. Bacterial isolation and antimicrobial susceptibility testing were performed, and phenotypic and genotypic detection of extended-spectrum β-lactamases (ESBLs) and cephamycins (AmpC) was carried out, as well as multidrug resistance (MDR) was calculated. Enterobacterales were detected in 92.1% CLH and in 100% FRH. *Escherichia coli* was the most frequent isolate, followed by *Klebsiella* spp. CLH showed higher MDR indices (45.4% with MAR ≥ 0.2) and a higher prevalence of resistance genes, including *bla*_CTX-M-1_, *bla*_CTX-M-2_, *bla*_CTX-M-8_, and *bla*_CMY-2_. In FRH, MDR was less frequent (18% with MAR ≥ 0.2), but resistance genes were also detected. In Caatinga biome conditions, CLH demonstrated higher frequency of carrying resistant bacteria, reflecting the intensive management of commercial poultry farming. However, FRH, through contact with the environment and other animals, also represent potential reservoirs of MDR Enterobacterales and associated genes.

## Introduction

Antimicrobial resistance (AMR) is considered a major global health challenge, particularly in low- and middle-income countries. It is estimated that approximately 4.95 million deaths worldwide were associated with bacterial resistance in 2019 [1]. AMR results from complex dissemination mechanisms involving the human, animal, and environmental interfaces, constituting a serious One Health issue intensified by the indiscriminate use of antimicrobials in human medicine, veterinary medicine, and food production [2].

Poultry farming is a widespread practice worldwide, and the high demand for poultry products due to their affordable cost drives global production [3]. Consequently, antimicrobial use in production systems has also increased, favoring the selection of resistant bacteria [4–6]. In intensive production systems, antimicrobials are frequently used not only to prevent or treat infections that may cause economic losses, but also as growth promoters [7, 8]. Although the use of these drugs is lower in free-range or extensive systems, greater interaction with the environmental resistome and other animal species may favor the acquisition of resistant bacteria and resistance genes [9, 10].

The isolation of extended-spectrum β-lactamase (ESBL) and AmpC-producing Enterobacterales has already been reported in broiler chickens [5, 11, 12]. These findings are equally concerning in other production systems, such as commercial laying hens and free-range laying hens. Recent data have revealed high multidrug resistance (MDR) rates in these flocks, reaching approximately 64.6% in commercial laying hens and 95.7% in organic systems, reinforcing that these birds and their products may act as reservoirs and vehicles for the dissemination of antimicrobial resistance throughout the food chain [13, 14].

Brazil is the world leader in chicken meat exports and the third largest producer globally [15]. In addition, most national microbiological studies have focused on broiler chickens, especially in regions with higher production rates, such as the South and Southeast regions [16–18]. However, egg production in 2025 reached 62.3 billion units, with an average consumption of 288 eggs per inhabitant, highlighting the importance of laying hens in this scenario [15].

The dynamics of AMR are complex and influenced by climatic and environmental factors [19]. In Northeastern Brazil, the Caatinga biome is characterized by high solar radiation, elevated soil temperatures, and intense cycles of drought and rainfall. This abiotic stress promotes intense competition for resources, selecting microbial communities adapted to extreme conditions. Such adaptations may include resistance mechanisms as survival strategies, reinforcing the relevance of understanding the regional antimicrobial resistance profile [20–22].

The lack of recent data regarding the antimicrobial susceptibility profile of bacteria isolated from laying hens in Brazil, combined with the scarcity of studies conducted in the Northeastern region, highlights the need for further investigations to better understand the dynamics of AMR dissemination in these sectors. Therefore, this study aimed to phenotypically and genotypically characterize the antimicrobial susceptibility profile of Enterobacterales isolated from commercial and free-range laying hens (*Gallus gallus domesticus*) raised under semiarid conditions.

## Materials and methods

### Study location, sampling and sample collection

This study was approved by the Ethics Committee on Animal Use (CEUA) of the Federal University of Campina Grande (UFCG) under protocol 67/2020. The CLH came from a farm in the state of Paraíba and two in the state of Rio Grande do Norte, both in the semiarid region of Northeastern Brazil. The FRH were obtained from five rural properties in the same states, raised in backyards, in contact with other birds such as ducks (*Domestic duck*) and guinea fowl (*Helmeted guineafowl*). The diet consisted of corn and food scraps, and the water came from local reservoirs. These properties were only for family consumption, with the surplus being sold in local markets. For CLH, the flock sizes varied from 2,000 to 3,000 animals aging 70–75 weeks, whereas for FRH, the flock sizes varied from 10 to 50 animals aging 24–36 months. In both systems, only therapeutic use of antimicrobials was adopted.

The minimum sample size was calculated using the formula for simple random samples, considering 95% confidence, expected frequency of 50% for sample maximisation and sampling error of 10% [23], resulting in 96 animals. However, cloacal swabs were collected from 165 CLH (September/2019 to February/2020) and 165 FRH (February to August/2020). The amount of swabs taken per flock was not equal because the fieldwork depended on the owner’s availability and the routine of the production unit.

Samples were packed into tubes with Stuart transport medium (Interlab; São Paulo, São Paulo, Brazil) and transported under refrigeration (2–8 °C) to the microbiology laboratory for processing within 24 h after collection.

### Isolation and bacterial identification

The samples were enriched in Brain Heart Infusion broth (KASVI; São José dos Pinhais, Paraná, Brazil) at 37 °C for 24 h and seeded on MacConkey agar (KASVI; São José dos Pinhais, Paraná, Brazil), with incubation under aerobic conditions at 37 °C for 24–48 h. After incubation, colonies displaying morphology compatible with Enterobacterales were selected. When more than one colony morphotype was observed on a plate, representative colonies of each distinct morphotype, based on differences in size, shape, elevation, margin, and lactose fermentation characteristics, were selected for further analysis. A maximum of two or three colonies per sample were analyzed. The selected colonies were evaluated by Gram staining and subjected to biochemical identification using Rugai medium (NEWPROV; Pinhais, Paraná, Brazil), allowing the simultaneous evaluation of glucose and sucrose fermentation, gas and H₂S production, urease, indole, lysine decarboxylation, and motility [24]. Complementary tests included TSI (Triple Sugar Iron), malonate, gelatinase, phenylalanine deaminase, citrate, methyl red (MR), Voges–Proskauer (VP), esculin, nitrate reduction, oxidase, and coagulase [25].

### Antimicrobial susceptibility test and phenotypic test for β-lactamases

The isolated bacteria were subjected to the susceptibility test in Müeller-Hinton agar by the disk-diffusion method (Kirby-Bauer) [26], according to recommendations from the Brazilian Committee on Antimicrobial Susceptibility Testing (BrCAST) [27] and the Clinical and Laboratory Standards Institute (CLSI) [28]. Moreover, the veterinary CLSI was used to determine the cut-off point for the antimicrobial enrofloxacin [29]. The strain of *Escherichia coli* CIP 76.24 (ATCC 25922) was used as quality control. The production of ESBL was inferred by the presence of deformation or a ghost zone between aztreonam (30 µg), ceftazidime (30 µg), cefotaxime (30 µg) and/or cefepime (30 µg) disks towards amoxicillin/clavulanate (20/10 µg) [27]. Suspected AmpC-type β-lactamase production was assessed by the cefoxitin (30 µg) inhibition halo [30].

MDR was assessed by the MAR index (ratio between antimicrobials to which the isolate showed resistance and the number of antimicrobials tested), considering isolates with MAR ≥ 0.2 to be MDR. For antimicrobial classes, the MCAR index was used (ratio between the classes of antimicrobials to which the isolate showed resistance and number of classes tested), considering MDR when MCAR ≥ 0.25. Isolates resistant to at least three classes were also classified as MDR [31, 32].

The following classes of antibiotics were evaluated: aminoglycosides: amikacin (30 µg), gentamicin (10 µg); fluoroquinolones: enrofloxacin (5 µg), norfloxacin (10 µg); phenicols: chloramphenicol (30 µg); tetracyclines: tetracycline (30 µg); third-generation cephalosporins: cefotaxime (30 µg), ceftriaxone (30 µg), ceftazidime (30 µg), ceftiofur (30 µg); carbapenems: ertapenem (10 µg), imipenem (10 µg), meropenem (10 µg); aminopenicillins: ampicillin (10 µg); aminopenicillin combined with a β-lactamase inhibitor: amoxicillin/clavulanate (20/10 µg); cefamicin: cefoxitin (30 µg); fourth-generation cephalosporins: cefepime (30 µg); monobactam: aztreonam (30 µg).

### Resistance gene identification

The DNA of the isolated Enterobacterales was extracted by the boiling method [33]. The detection of ESBL and AmpC genes was performed by polymerase chain reaction (PCR) kit (ThermoFisher Scientific Brazil; São Paulo, São Paulo, Brazil). The genes *bla*_CTX−M_ (groups 1, 2, 9 and 8/25) and *bla*_CMY−2_ were identified by multiplex and conventional PCR, as described by Dallenne et al. [34] and Mammeri et al. [35], respectively (Table 1).

**Table 1:** Sequence of primers used to detect genes responsible for the production of ESBL and AmpC in Enterobacterales isolates from laying hens (CLH) and free-range hens (FRH) from Caatinga biome

Type of PCR	Target group	Primer	Sequence (5’→3’)	Product	Reference
Multiplex CTX-M-1/2/9	*bla*_CTX-M_ / group 1	Forward Reverse	TTAGGAARTGTGCCGCTGYA CGATATCGTTGGTGGTRCCAT	688 bp	[34]
Multiplex CTX-M-1/2/9	*bla*_CTX-M_ / group 2	Forward Reverse	CGTTAACGGCACGATGAC CGATATCGTTGGTGGTRCCAT	404 bp	
Multiplex CTX-M-1/2/9	*bla*_CTX-M_ / group 9	Forward Reverse	TCAAGCCTGCCGATCTGGT TGATTCTCGCCGCTGAAG	561 bp	
Multiplex CTX-M-8/25	*bla*CTX-_M-8, -25, -26, -39 -41_	Forward Reverse	AACRCRCAGACGCTCTAC TCGAGCCGGAASGTGTYAT	326 bp	
Conventional	*bla*_CMY-2_	CMY-2-A	5’-ATGATGAAAAAATCCTTATCC-3’	-	[35]
Conventional	*bla*_CMY-2_	CMY-2-B	5’-TTATTCGACCTTTTCAAGAATGC-3’	-	
Conventional	*bla*_ACT-1_	ACT-1-A	5’-ATGATGACTAAATCCCTTTGC-3’	-	
Conventional	*bla*_ACT-1_	ACT-1-B	5’-CTACACGCCCGCTCAAATACG-3’	-	
Conventional	*bla*_ACC-1_	ACC-1-A	5’-ATGCAGAACACATTGAAGC-3’	-	
Conventional	*bla*_ACC-1_	ACC-1-B	5’-CTACTTATTCCCTTCCAATGAGC-3’	-	
Conventional	*bla*_DHA-1_	DHA-1-A	5’-ATGAAAAATCGTTATCTCC-3’	-	
Conventional	*bla*_DHA-1_	DHA-1-B	5’-TTATTCCAGTGCACTCAAAATAGC-3’	-	
Conventional	*bla*_FOX-1_	FOX1-1-A	5’-ATGCAACAACGACCTGCGTTCG-3’	-	
Conventional	*bla*_FOX-1_	FOX1-1-B	5’-TCACTCGGCCAACTGACTCAGG-3’	-	

### Statistical analysis

To compare the frequencies of bacteria isolated from chicken cloacae, the chi-squared goodness-of-fit test was used, and the comparison of antimicrobial resistance frequency between CLH and FRH the independence chi-squared test was used. The significance level of 5% was deemed, and the analyses were run in the R environment and RStudio interface [36].

## Results

Of the 165 CLH analysed, 152 (92.1%) showed growth of Enterobacterales, whereas all 165 FRH were positive. In the latter, 37 samples showed more than one type of colony, totalling 198 isolates. In both groups, *Escherichia coli* was the most frequent agent (73.7% in CLH and 68.2% in FRH), followed by *Klebsiella* spp. (13.2% and 12.6%, respectively). A statistical difference was observed (*P* < 0.0001) between the frequencies of bacteria isolated in both laying hens and free-range animals. Other species were also present, but at a lower frequency (Table 2).

**Table 2:** Frequency of bacterial isolates among commercial and free-range laying hens from Caatinga biome

Bacteria	Laying hens (%)	Free-range hens (%)
*Escherichia coli*	112 (73.7)	135 (68.2)
*Klebsiella* spp.	20 (13.16)	25 (12.6)
*Proteus mirabilis*	8 (5.26)	1 (0.5)
*Salmonella* spp.	5 (3.29)	7 (3.6)
*Edwardsiella* spp.	4 (2.63)	20 (10.1)
*Citrobacter* spp.	1 (0.66)	6 (3)
*Providencia* spp.	1 (0.66)	–
*Shigella* spp.	1 (0.66)	–
*Enterobacte*r spp.	–	4 (2)
**Total**	152 (100)	198 (100)

The highest resistance rates in CLH were observed for tetracycline (59.21%), ampicillin (56.58%), norfloxacin (42.76%), enrofloxacin (42.11%), gentamicin (22.37%), ceftriaxone (21.05%) and ertapenem (17.76%), with no resistance detected to imipenem and meropenem. In FRH, isolated bacteria showed resistance mainly to tetracycline (44.4%), ampicillin (28%), amoxicillin/clavulanic acid (21%), cefepime (20.5%), ertapenem (16.5%) and ceftriaxone (14.5%). MDR indices varied between the groups studied. In CLH, the MAR values ranged from 0 to 0.67 (average 0.19), with 69 (45.4%) of the isolates showing MAR ≥ 0.2. In FRH, the values ranged from 0 to 0.94 (average 0.12), and 36 (18%) isolates with MAR ≥ 0.2. As for the MCAR index, in CLH the values ranged from 0 to 0.82 (average 0.24), with 74 (48.7%) of the isolates ≥ 0.25; in contrast, in FRH they ranged from 0 to 1 (average 0.16), with 25% of the isolates ≥ 0.25 (Table 3).

**Table 3:** MAR and MCAR indices among isolates from commercial laying and free-range hens from Caatinga biome

Bacteria	Laying hens	Laying hens	Laying hens	Laying hens	Free-range hens	Free-range hens	Free-range hens	Free-range hens
	(MAR ≥ 0.2)	Average MAR	(MCAR ≥ 0.25)	Average MCAR	(MAR ≥ 0.2)	Average MAR	(MCAR ≥ 0.25)	Average MCAR
*Escherichia coli*	57	0.34	59		0.41	22	0.43	31
*Klebsiella* spp.	10	0.54	12		0.57	3	0.30	4
*Salmonella* spp.	1	0.56	2		0.46	2	0.28	3
*Edwadsiella* spp.	1	0.28	1		0.36	6	0.48	8
*Citrobacter* spp.	-	-	-		-	1	0.28	2
*Enterobacter* spp.	-	-	-		-	1	0.22	1

In CLH, 14 isolates of *E. coli*, eight *Klebsiella* spp. and one *Salmonella* spp. were positive for phenotypic expression of ESBL, in addition to 10 *E. coli* and one *Klebsiella* spp. for AmpC. One isolate of *Klebsiella* spp. showed simultaneous phenotypic positivity for ESBL and AmpC. In the genotypic test, the genes *bla*_CTX−M−1−Like_ were detected in four *E. coli* and four *Klebsiella* spp., *bla*_CTX−M−2−Like_ in one *Salmonella* spp., two *E. coli* and three *Klebsiella* spp., *bla*_CTX−M−8−Like_ in six *E. coli* and *bla*_CMY−2−Like_ in nine *E. coli* and two *Klebsiella* spp. In FRH, 13 isolates of *E. coli* and one of *Klebsiella* spp. were positive for the ESBL phenotype, whereas six *E. coli*, one *Klebsiella* spp. and one *Salmonella* spp. showed positivity for AmpC. As for the genotypic profile, the genes *bla*_CTX−M−1−Like_, *bla*_CTX−M−2−Like_ and *bla*_CMY−2−Like_ were detected in three isolates of *E. coli* (Table 4). However, there was no significant difference between CLH and FRH in terms of antimicrobial resistance (*P* = 0.341).

**Table 4:** Detection of resistance genes by multiplex/conventional PCR in Enterobacterales isolates from commercial laying and free-range hens from Caatinga biome

Genes	Bacteria	Number of positive isolates	Origin	Total number of isolates
*bla*_CTX−M1−like_	*Escherichia coli*	4	Laying hens	152
	*Klebsiella* spp.	4	Laying hens	152
*bla*_CTX−M2−like_	*Escherichia coli*	2	Laying hens	152
	*Klebsiella* spp.	3	Laying hens	152
	*Salmonella* spp.	1	Laying hens	152
*bla*_CTX−M8−like_	*Escherichia coli*	6	Laying hens	152
	*Klebsiella* spp.	1	Laying hens	152
*bla*_CMY−2−like_	*Escherichia coli*	9	Laying hens	152
	*Klebsiella* spp.	2	Laying hens	152
*bla*_CTX−M1−like_	*Escherichia coli*	1	Free-range hens	198
*bla*_CTX−M2−like_	*Escherichia coli*	1	Free-range hens	198
*bla*_CMY−2−like_	*Escherichia coli*	1	Free-range hens	198

## Discussion

The isolation rate of Enterobacterales in commercial laying hens (CLH) was 92.1%, whereas all free-range hens (FRH) were colonized by these organisms. Although they are part of the normal intestinal microbiota of birds, the high isolation rate observed reinforces the role of these animals as important reservoirs of Enterobacterales throughout the poultry production chain and at the human–animal–environment interface [37]. The high positivity observed in FRH, including samples exhibiting growth of multiple colonies, highlights the influence of extensive management practices on the diversified composition of the intestinal microbiota of these animals, such as unrestricted feeding behavior, increased exposure to open environments, and contact with synanthropic fauna [38–42].

*E. coli* was the most frequently identified species in both CLH and FRH samples. This predominance extends beyond its commensal role, since pathogenic strains such as avian pathogenic *E. coli* (APEC), the etiological agent of colibacillosis, constitute an important epidemiological, economic, and animal welfare concern. This pathogen is considered one of the main drivers of antimicrobial use in poultry flocks and is associated with high levels of antimicrobial resistance [43, 44]. Furthermore, other zoonotically relevant pathotypes, such as enterotoxigenic *E. coli* (ETEC) and Shiga toxin-producing *E. coli* (STEC), are involved in foodborne outbreaks, reinforcing the importance of microbiological surveillance in poultry products [45].

The genus *Klebsiella* was the second most prevalent in both groups. Although it is part of the intestinal microbiota of birds, under conditions of stress and immunosuppression it may cause opportunistic infections mediated by complex virulence mechanisms, such as iron acquisition systems, resulting in mortality and economic losses [46, 47]. Recent studies have shown that *Klebsiella pneumoniae* subsp. *pneumoniae* has a significant prevalence in the intestinal tract of healthy birds, including broilers and laying hens [48, 47]. The identification of this pathogen in healthy humans [49] further reinforces the human–animal interface and suggests the importance of poultry and poultry products in the dissemination of *K. pneumoniae* through the food chain, owing to its remarkable ability to adapt to different ecological niches.

Despite the low isolation frequency observed in CLH and FRH, the detection of *Salmonella* spp. is of considerable epidemiological and zoonotic relevance, as its presence in healthy birds may represent a continuous risk to public health due to its capacity for vertical transmission (via eggs) and persistence within production systems [50–52]. Salmonellosis is the second most commonly reported gastrointestinal infection in humans and is mainly associated with contamination of eggs and egg-derived products by *Salmonella enteritidis*, highlighting laying hens as important sources of infection [53]. In Brazil, *Salmonella* spp. have been recovered from healthy commercial broiler flocks and table eggs, demonstrating their virulence and ability to disseminate resistance determinants through food products, making these birds potential silent reservoirs of antimicrobial resistance [54, 55].

Other members of the order Enterobacterales, such as *Proteus mirabilis*, *Edwardsiella* spp., *Citrobacter* spp., and *Enterobacter* spp., were also detected at lower frequencies in CLH and FRH. The presence of these organisms supports the hypothesis that poultry production environments act as diverse ecological reservoirs for opportunistic and potentially zoonotic pathogens [56, 57, 58]. In southern Brazil, *P. mirabilis* isolates recovered from chicken meat showed 100% genetic similarity to isolates obtained from human urinary tract infections, demonstrating their potential for community dissemination through poultry products [56].

The detection of *Edwardsiella* spp., *Citrobacter* spp., and *Enterobacter* spp. may be associated with common environmental exposure and the adaptive plasticity of these microorganisms across different habitats, particularly the genus *Edwardsiella*, which circulates between aquatic environments and animal hosts, including birds and humans [59, 60]. Similarly, the occurrence of *Citrobacter* spp. and *Enterobacter* spp. reinforces the need for surveillance of poultry products because of their ability to acquire and disseminate resistance determinants throughout the food chain, particularly *Enterobacter* spp., owing to their efficiency in penetrating eggshell membranes and compromising the microbiological quality of eggs for human consumption [58, 61, 62].

CLH isolates exhibited high rates of resistance to tetracyclines, aminopenicillins, and quinolones, whereas resistance to tetracyclines and β-lactams predominated among FRH isolates. Although the use of these compounds as growth promoters and performance-enhancing feed additives in food-producing animals was prohibited in Brazil in 2009 [63], high levels of resistance are still observed among poultry-associated isolates in the country [64].

The persistence of resistance in poultry production systems, particularly conventional operations, may be associated with the historical use of these compounds in animal production and the ecological maintenance of resistance determinants, a phenomenon further intensified by the continued availability of these antimicrobials for therapeutic and prophylactic use [58]. Moreover, antimicrobial residues detected in poultry muscle tissues and litter may actively contribute to the maintenance of AMR within the human–animal–environment interface through food products for human consumption and the reuse of poultry waste as agricultural fertilizer and, historically, as cattle feed [65–67].

Multidrug resistance (MDR) rates were higher among CLH isolates, particularly in *E. coli* and *Klebsiella* spp., corroborating national and international findings which showed higher frequency of MDR Enterobacterales in intensive production systems compared with extensive systems [64, 68, 69]. This pattern may be related to the characteristics of intensive poultry farming, where high stocking densities favor the occurrence of infectious diseases [70] and consequently increase the use of antimicrobials for therapeutic, prophylactic, or metaphylactic purposes. This scenario generates continuous selective pressure on the avian microbiota, contributing to the maintenance of resistance both within and beyond production systems [71, 72].

Despite the lower MDR rate observed in the FRH group, the high prevalence of MDR in *Salmonella* spp. and *Edwardsiella* spp. also represents a warning for food safety and One Health initiatives in Brazil. Multidrug resistance in poultry-associated *Salmonella* spp. is of particular epidemiological concern because its detection in apparently healthy birds reflects the risk of foodborne disease outbreaks while simultaneously contributing to the dissemination of clinically relevant resistance determinants through the food chain [54, 55].

The detection of MDR *Edwardsiella* spp. in the FRH system may be intrinsically associated with the animal–environment interface, since these birds shared their environment with ducks and guinea fowl and had close contact with pond water. In the semiarid region of northeastern Brazil, due to water scarcity, such natural reservoirs are commonly used for fish farming, especially tilapia production [73]. Furthermore, *Edwardsiella tarda* has previously been isolated from ducks and tilapia inhabiting a natural lake in southeastern Brazil and was associated with an outbreak of acute mortality in these animals. The high genetic similarity observed among isolates reinforces the ability of this bacterium to cross interspecies barriers and highlights the importance of water quality management in animal production systems [74].

Although identified at lower frequencies in both groups (CLH and FRH), resistance to β-lactam antibiotics deserves particular attention because of the epidemiological relevance of the resistance determinants detected. CTX-M groups 1, 2, and 8 were predominantly identified among CLH isolates, whereas only two FRH isolates were positive for groups 1 and 2. These findings are consistent with the national epidemiological profile, in which CTX-M-2, CTX-M-15, and CTX-M-8 are the most widespread variants in Brazil [75]. Although CTX-M-1 is epidemiologically well established in Europe and in animal production systems, its variant blaCTX-M-15 has achieved worldwide dissemination and is particularly prevalent in human isolates [76, 77].

This interconnectivity is further reinforced by the detection of the blaCTX-M-8 gene in fecal samples from healthy food handlers and in chicken meat imported from Brazil into Japan, where genetically related isolates were identified, highlighting its international impact [78]. These findings support the potential role of Brazilian poultry products in the dissemination of antimicrobial resistance determinants at both transregional and international levels and help address important gaps in microbiological surveillance data from northeastern Brazil, where epidemiological information remains scarce compared with the southern and southeastern regions of the country [75].

The blaCTX-M gene family represents the most widespread ESBL determinant worldwide and, because it is frequently associated with MDR genetic platforms, it contributes to the maintenance of multidrug resistance through co-selection mechanisms [79]. This phenomenon allows the preservation of multiple resistance genes within a bacterial host under selective pressure exerted by a single antimicrobial agent, heavy metals, mineral supplements, or disinfectants. Consequently, multidrug resistance may persist in animal production systems even after regulatory restrictions on the use of specific antimicrobials as growth promoters [55, 56, 76].

The blaCMY-2-like gene was also more frequently detected among CLH isolates, whereas only one positive isolate was identified among FRH. Although ampC genes may be located on the chromosome of certain Enterobacterales species, their expression levels are generally lower than those associated with plasmid-mediated variants, favoring the rapid dissemination of the latter [80]. The prevalence of this gene varies according to host species and geographical region and has historically been more significant in North America among both humans and food-producing animals. However, since the first reports of blaCMY-2 in human isolates in Brazil, this gene has been widely detected in food products, particularly poultry products, reinforcing the role of Brazilian poultry production as a reservoir and potential source of dissemination of resistance genes [81–83].

In this context, the occurrence of ESBL- and/or AmpC-producing Enterobacterales in Brazilian poultry production systems represents a major public health challenge because it limits therapeutic options and increases dependence on critically important antimicrobials for human medicine, such as carbapenems [79, 84]. Although resistance to this class was detected at low frequencies in both CLH and FRH isolates, which is consistent with most regional studies reporting low carbapenem resistance levels among poultry isolates [85–87], its detection should be regarded as an important epidemiological warning.

The detection of resistance to ertapenem suggests the involvement of non-enzymatic resistance mechanisms associated with prior ESBL and/or AmpC production, such as porin loss and active efflux, since the presence of carbapenemases would generally confer resistance to the entire carbapenem class [80]. These findings reinforce the interconnectedness of the human–animal–environment interface in the context of AMR, especially considering that the use of these antibiotics is highly restricted in veterinary medicine [88]. Although carbapenem resistance remains uncommon among poultry isolates in northeastern Brazil, recent studies have reported resistance rates of 63.3% to imipenem and 59.1% to ertapenem among isolates recovered from eggs produced by free-range chickens in the semiarid region [62]. These results indicate a heterogeneous and epidemiologically critical resistome in northeastern Brazil that varies according to management practices and ecological niches, highlighting the need for continuous monitoring of the poultry production chain to mitigate the expansion of these resistance mechanisms.

β-lactamase-producing *E. coli* and *Klebsiella* spp. constitute a major challenge for both human and animal health and are classified by the World Health Organization as critically important priority pathogens [89]. Phenotypic and genotypic expression of ESBL and AmpC was more frequently observed among CLH isolates, particularly in *E. coli* and *Klebsiella* spp., reflecting the established epidemiological profile of the major poultry-producing regions of Brazil. Studies carried out in southern and southeastern Brazil have demonstrated the circulation of ESBL/AmpC-producing *E. coli* and *K. pneumoniae* in chicken meat, cloacal swabs, poultry litter, and human samples, associated with the presence of blaCTX-M-2, blaCTX-M-15, blaCTX-M-55, and blaCMY-2 genes and frequently exhibiting multidrug-resistant phenotypes [90, 16, 17, 18].

Investigations into the occurrence of ESBL/AmpC-producing Enterobacterales throughout the poultry production chain remain scarce in northeastern Brazil. Available reports have primarily focused on the detection of these microorganisms in eggs [62], broilers raised on commercial farms [87], and domestic birds such as chickens and ducks [85]. Therefore, the identification of MDR isolates carrying clinically relevant resistance genes in different poultry production systems (CLH and FRH) expands current knowledge of the epidemiological context of antimicrobial resistance in the Brazilian poultry sector and underscores the need for microbiological surveillance from a One Health perspective throughout the country.

## Conclusion

The results of this study demonstrated that CLH exhibit major frequency of carrying bacteria with relevant resistance determinants due to intensive production management. Although FRH isolates exhibit lower resistance rates, these animals can acquire and disseminate bacteria and resistance genes through contact with the environment, untreated water, and other animal species. These findings reinforce the importance of surveillance in the poultry sector, considering that Brazil is among the world’s largest producers and exporters of chicken. The presence of resistant bacteria in poultry and their products can favor the spread of AMR, thus affecting humans, animals, and the environment, constituting a serious One Health problem.

## Data Availability

Data are available on request from the corresponding author.
